# Supplementary material for: Does frequency or diversity of leisure activity matter more for epigenetic ageing? Analyses of arts engagement and physical activity
Source: medRxiv. 2025 Sep 16:2024.11.01.24316559. Preprint. [Version 2] doi: 10.1101/2024.11.01.24316559 (PMC12458519; doi:10.1101/2024.11.01.24316559)
Supplement: 1 [file NIHPP2024.11.01.24316559V2-supplement-1.pdf]

medRxiv preprint doi: https://doi.org/10.1101/2024.11.01.24316559; this version posted September 16, 2025. The copyright holder for this preprint (which was not certified by peer review) is the author/funder, who has granted medRxiv a license to display the preprint in perpetuity.

It is made available under a CC-BY 4.0 International license .

Supplementary Materials

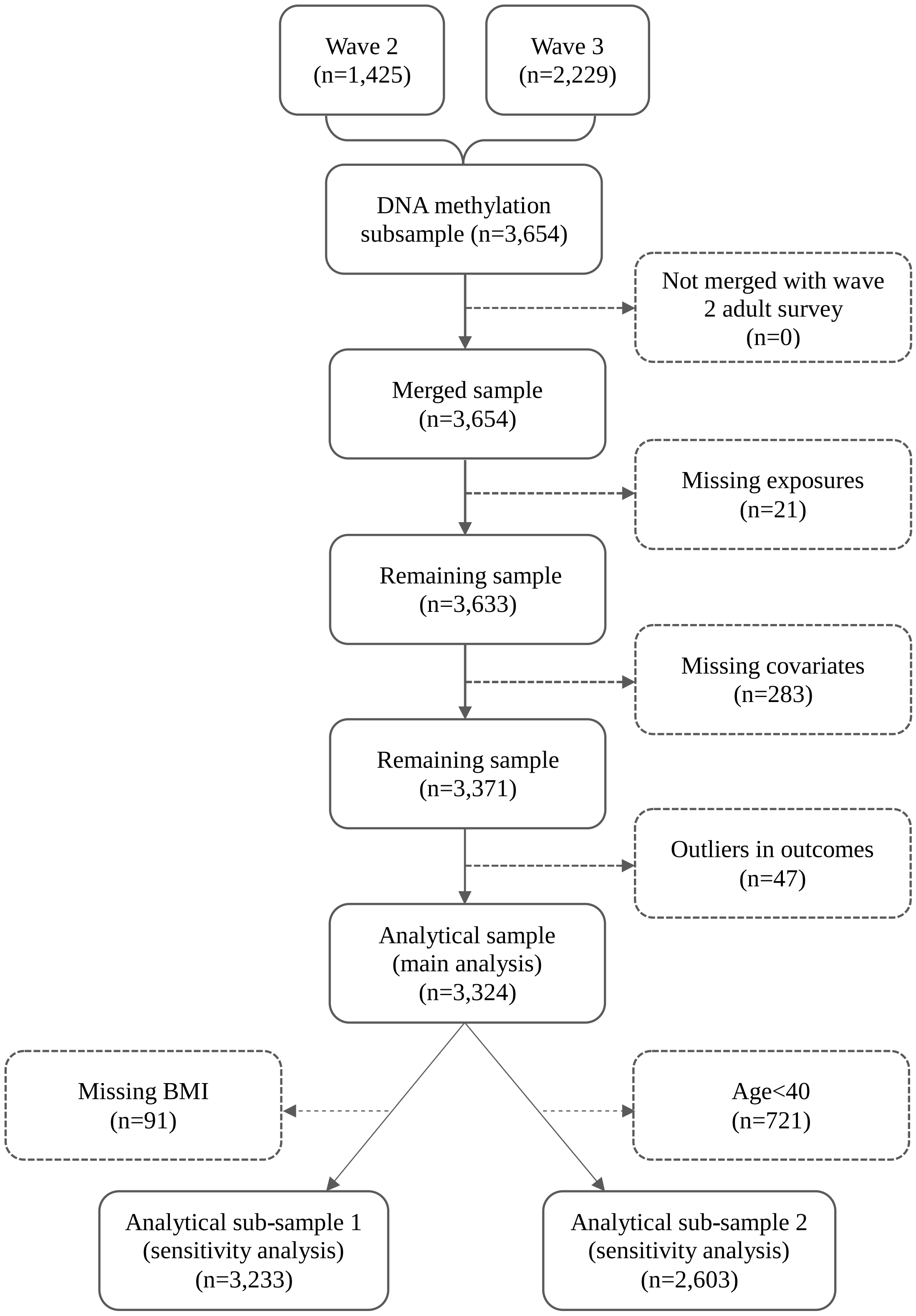

Figure S1. Sample selection diagram

medRxiv preprint doi: https://doi.org/10.1101/2024.11.01.24316559; this version posted September 16, 2025. The copyright holder for this preprint (which was not certified by peer review) is the author/funder, who has granted medRxiv a license to display the preprint in perpetuity.

It is made available under a CC-BY 4.0 International license .

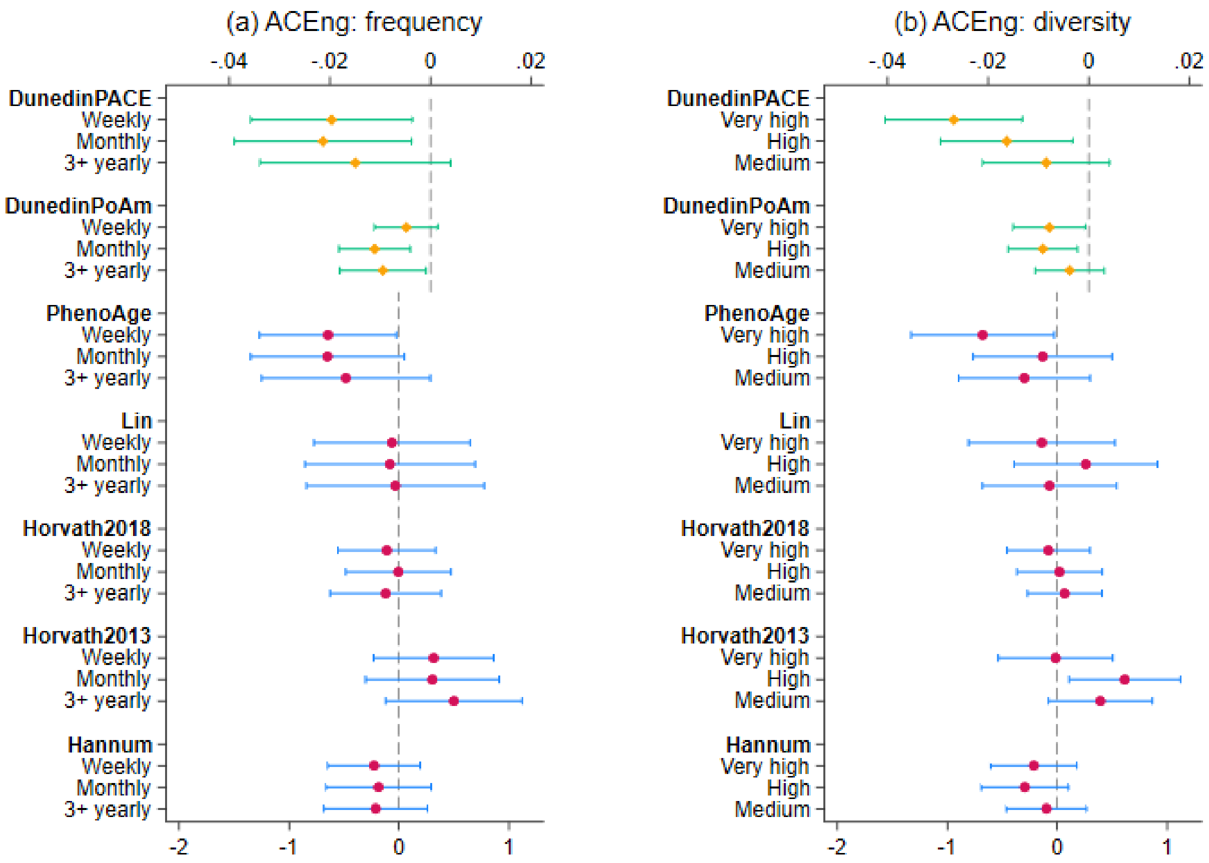

Figure S2. Estimated average treatment effect and 95% confidence intervals for ACEng diversity and frequency measures from doubly robust estimation using IPWRA (additionally controlling for BMI, n=3,233)

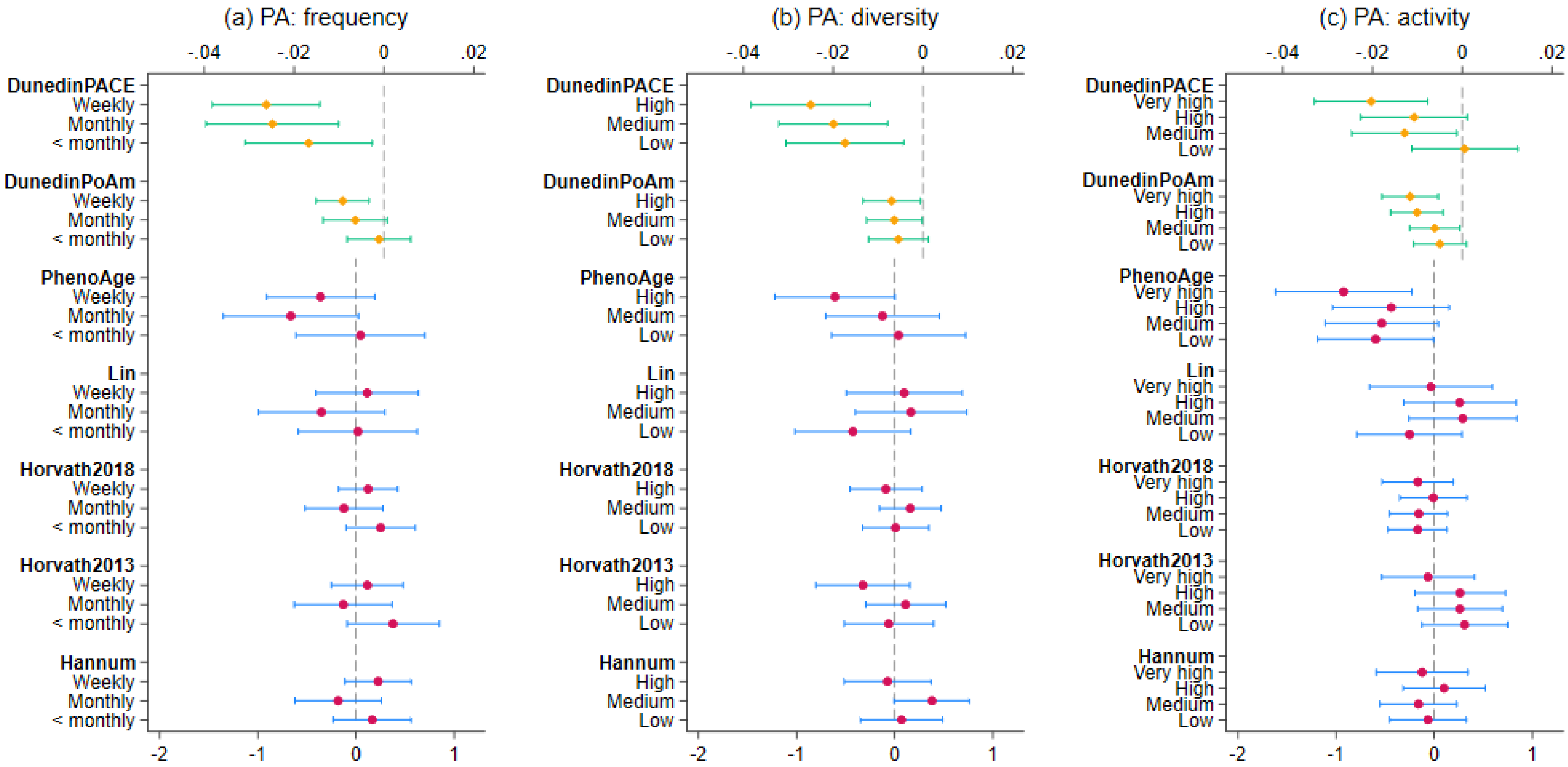

Figure S3. Estimated average treatment effect and 95% confidence intervals for PA diversity, frequency and activeness from doubly robust estimation using IPWRA (additionally controlling for BMI, n=3,233)

medRxiv preprint doi: https://doi.org/10.1101/2024.11.01.24316559; this version posted September 16, 2025. The copyright holder for this preprint (which was not certified by peer review) is the author/funder, who has granted medRxiv a license to display the preprint in perpetuity.
It is made available under a CC-BY 4.0 International license .

medRxiv preprint doi: https://doi.org/10.1101/2024.11.01.24316559; this version posted September 16, 2025. The copyright holder for this preprint (which was not certified by peer review) is the author/funder, who has granted medRxiv a license to display the preprint in perpetuity.

It is made available under a CC-BY 4.0 International license .

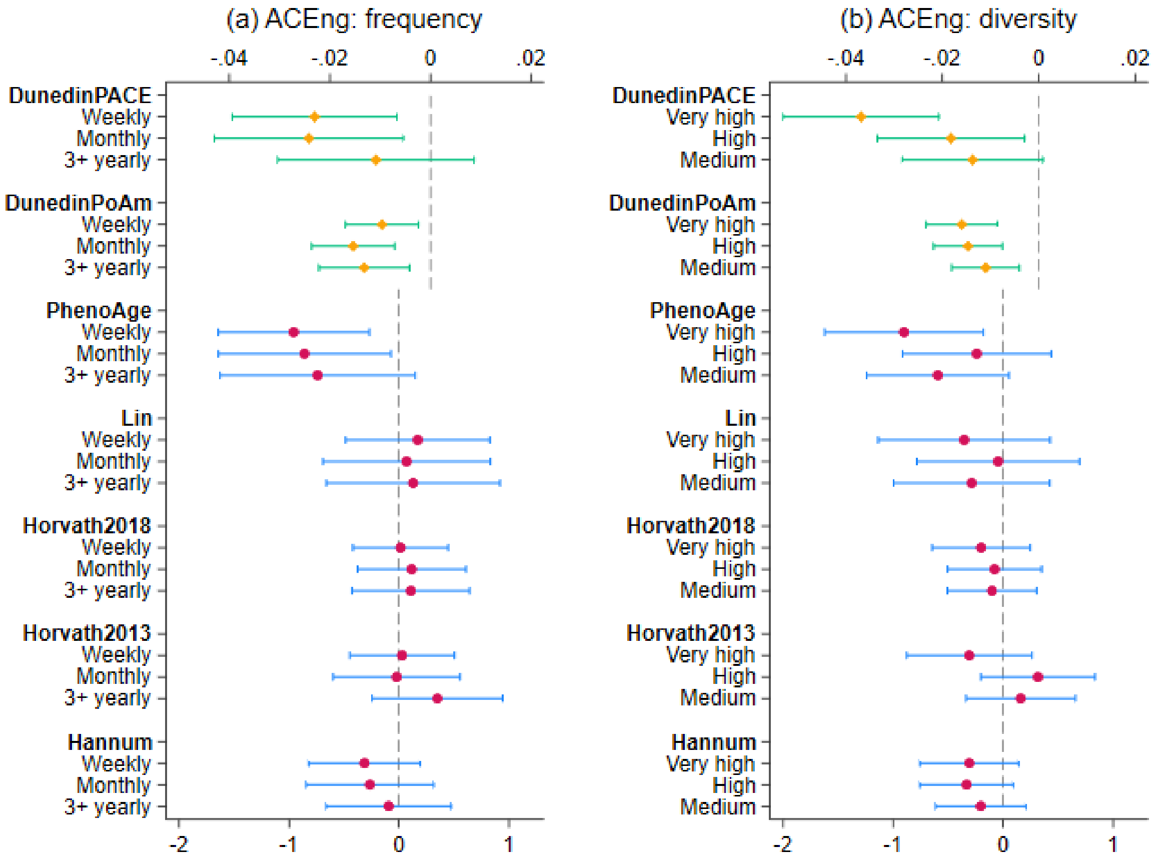

Figure S4. Estimated average treatment effect and 95% confidence intervals for ACEng diversity and frequency measures from doubly robust estimation using IPWRA (age>=40, n=2,603)

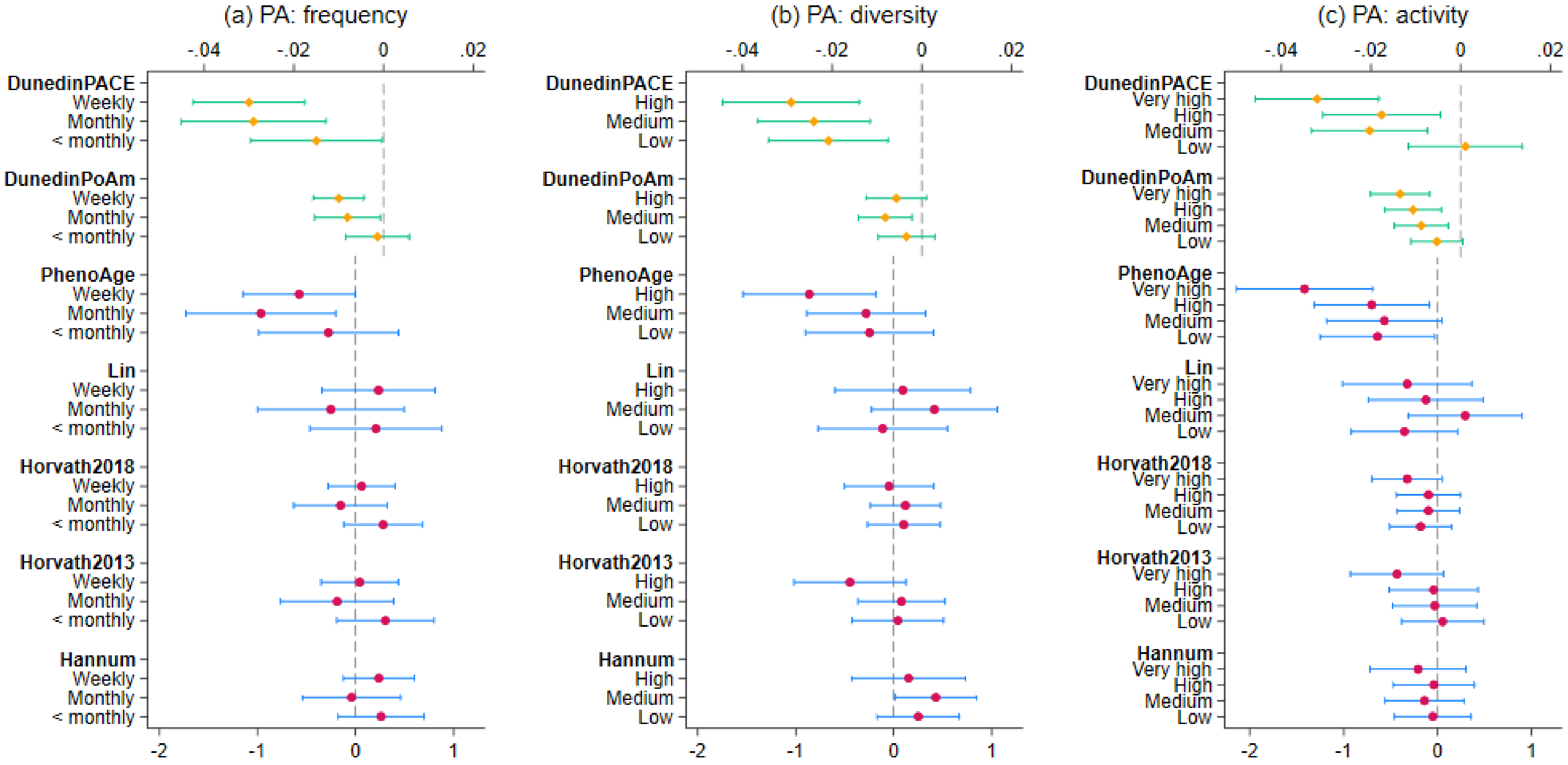

Figure S5. Estimated average treatment effect and 95% confidence intervals for PA diversity, frequency and activeness from doubly robust estimation using IPWRA (age>=40, n=2,603)

medRxiv preprint doi: https://doi.org/10.1101/2024.11.01.24316559; this version posted September 16, 2025. The copyright holder for this preprint (which was not certified by peer review) is the author/funder, who has granted medRxiv a license to display the preprint in perpetuity.
It is made available under a CC-BY 4.0 International license .
